# A rare case of Intracavitary right ventricular cavernous lymphangioma in an adult: imaging–pathologic correlation

**DOI:** 10.1093/ehjcr/ytag169

**Published:** 2026-03-13

**Authors:** Xiaobei Shi, Kai Yang, Minjie Lu

**Affiliations:** Department of Magnetic Resonance Imaging, Fuwai Hospital and National Center for Cardiovascular Diseases, Chinese Academy of Medical Sciences and Peking Union Medical College, Beilishi Road No. 167, Xicheng District, Bejing 100037, China; Department of Radiology, Hangzhou First People's Hospital, No.261 Huansha Road, Shangcheng District, Hangzhou, Zhejiang Province 310006, China; Department of Magnetic Resonance Imaging, Fuwai Hospital and National Center for Cardiovascular Diseases, Chinese Academy of Medical Sciences and Peking Union Medical College, Beilishi Road No. 167, Xicheng District, Bejing 100037, China; Department of Magnetic Resonance Imaging, Fuwai Hospital and National Center for Cardiovascular Diseases, Chinese Academy of Medical Sciences and Peking Union Medical College, Beilishi Road No. 167, Xicheng District, Bejing 100037, China

**Keywords:** Cardiac, Cavernous lymphangioma, CMR

A 40-year-old man was incidentally found to have a right ventricular mass during a routine health examination using ultrasound. He had no relevant medical history, and electrocardiographic findings were normal. Transthoracic echocardiography revealed a nodular, moderately echogenic mass within the right ventricular cavity. Subsequent chest computed tomography (CT) demonstrated a nodular lesion adjacent to the anterior wall of the right ventricle, accompanied by proximal bundle-like low-density shadows (Panel A). Further cardiac magnetic resonance (CMR) imaging showed a well-defined, pedunculated nodule attached to the subvalvular chordae of the tricuspid valve, measuring approximately 15 × 17 mm (Panel B). The lesion exhibited heterogeneous high signal intensity on T1-weighted fat-suppressed sequences (Panel C), high signal intensity on T2-weighted images (Panel D), and heterogeneous enhancement on delayed gadolinium-enhanced imaging (Panel E). Based on these imaging findings, the lesion was considered benign.

The patient subsequently underwent surgical resection of the mass. Intraoperatively, the lesion appeared bright red and closely resembled normal myocardial tissue. On sectioning, the tumour was cystic and contained both blood and clear, colourless fluid. Histopathological examination confirmed the diagnosis of cavernous lymphangioma. Over an 8-year postoperative follow-up period, the patient remained in good health with no evidence of tumour recurrence.

Cardiac lymphangioma in adults is an uncommon benign cardiac tumour, with intracavitary cavernous lymphangioma being particularly rare. Given its pedunculated attachment and heterogeneous enhancement pattern, this entity should be differentiated from other intracavitary and valvular lesions, including myxomas and hematocysts.

**Figure:**
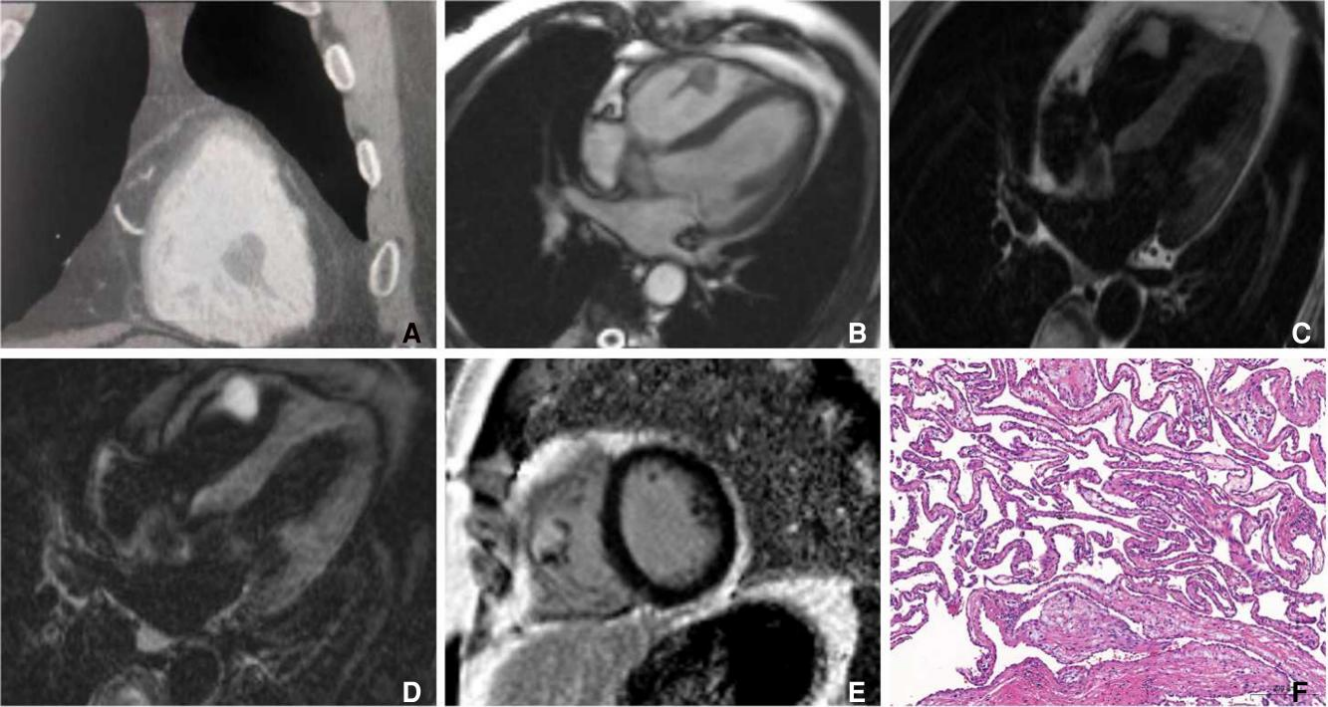


## Data Availability

The data underlying this article cannot be shared publicly due to patient privacy concerns. The data are available from the corresponding author upon reasonable request.

